# M402, a Novel Heparan Sulfate Mimetic, Targets Multiple Pathways Implicated in Tumor Progression and Metastasis

**DOI:** 10.1371/journal.pone.0021106

**Published:** 2011-06-16

**Authors:** He Zhou, Sucharita Roy, Edward Cochran, Radouane Zouaoui, Chia Lin Chu, Jay Duffner, Ganlin Zhao, Sean Smith, Zoya Galcheva-Gargova, Juliane Karlgren, Nancy Dussault, Rain Y. Q. Kwan, Erick Moy, Marishka Barnes, Alison Long, Chris Honan, Yi Wei Qi, Zachary Shriver, Tanmoy Ganguly, Birgit Schultes, Ganesh Venkataraman, Takashi Kei Kishimoto

**Affiliations:** Momenta Pharmaceuticals, Inc., Cambridge, Massachusetts, United States of America; Univesity of Texas Southwestern Medical Center at Dallas, United States of America

## Abstract

Heparan sulfate proteoglycans (HSPGs) play a key role in shaping the tumor microenvironment by presenting growth factors, cytokines, and other soluble factors that are critical for host cell recruitment and activation, as well as promoting tumor progression, metastasis, and survival. M402 is a rationally engineered, non-cytotoxic heparan sulfate (HS) mimetic, designed to inhibit multiple factors implicated in tumor-host cell interactions, including VEGF, FGF2, SDF-1α, P-selectin, and heparanase. A single *s.c.* dose of M402 effectively inhibited seeding of B16F10 murine melanoma cells to the lung in an experimental metastasis model. Fluorescent-labeled M402 demonstrated selective accumulation in the primary tumor. Immunohistological analyses of the primary tumor revealed a decrease in microvessel density in M402 treated animals, suggesting anti-angiogenesis to be one of the mechanisms involved *in-vivo*. M402 treatment also normalized circulating levels of myeloid derived suppressor cells in tumor bearing mice. Chronic administration of M402, alone or in combination with cisplatin or docetaxel, inhibited spontaneous metastasis and prolonged survival in an orthotopic 4T1 murine mammary carcinoma model. These data demonstrate that modulating HSPG biology represents a novel approach to target multiple factors involved in tumor progression and metastasis.

## Introduction

Metastatic cancer is a complex disease comprised of multiple microenvironments or niches involving the primary tumor, metastatic lesions, and the bone marrow [1]. These niches are interconnected by circulating tumor- and host-derived cells as well as soluble factors. Notably, many of the soluble factors implicated in progression of metastatic disease are currently targets for anti-cancer strategies. Tyrosine kinase inhibitors that inactivate pathways known to be important in certain cancers, such as chronic myeloid leukemia, renal cancer, gastrointestinal stromal tumor, have proven to have a profound effect on the clinical outcome,. However, in practice, the beneficial activity of such agents can be short-lived presumably due to mutations in the target or redundant pathways exploited by multiple tumor types. For example, anti-VEGF therapies, such as bevacizumab, can lead to tumors adaptation by exploiting alternative angiogenic factors and recruiting myeloid derived suppressor cells (MDSCs) to promote angiogenesis [2], resulting in resistance to these therapies and potentially the emergence of tumor cells with a more aggressive phenotype [3], [4]. To circumvent these shortcomings, there has been an increased effort in identifying combinations of targeted drugs that can be used together in cancer treatments. However, the combination of potent single-targeted drugs is often limited by their safety profile, and can result in unexpected adverse activity [5]. Despite these shortcomings, targeting extracellular, soluble factors has a number of significant benefits, including ease of delivery, and the ability to target multifaceted compartments, including the primary tumor, circulating tumor cells, as well as metastatic loci [6].

In an effort to design such an agent, we have focused on a unique class of molecules, the heparan sulfate proteoglycans (HSPGs). HSPGs are highly charged linear polysaccharides covalently bound to core proteins associated with the plasma membrane, such as syndecans and glypicans, or the basement membrane, such as perlecan, agrin, and collagen XVIII [7]. Many of the soluble factors that support tumor progression are HSPG-binding proteins [8]–[10] and this binding is known to modulate activity [9], [10]. For example, HSPG-bound growth factors, such as VEGF, FGF2, and HGF support the growth of new blood vessels. In addition, tumor cells utilize chemokines, such as SDF-1α, presented on HSPGs, to actively recruit host cells such as endothelial progenitor cells (EPCs) and MDSCs [11], [12] which have been shown to facilitate tumor progression in animal models and patients [13], [14]. HSPGs themselves can also serve as ligands for adhesion molecules such as P-selectin, which can mediate tumor-platelet and tumor-endothelial cell interactions [15] during dissemination of metastatic cells. Notably, HSPG-deficient tumor cells have been reported to exhibit severely impaired ability to form tumors *in-vivo* [16]. The convergence of multiple host- and tumor-derived factors on HSPGs makes them an attractive target for therapeutic intervention.

Using a systematic process to critically examine the structure-function relationships for heparan sulfate mimetics, we developed M402, a rationally engineered heparan sulfate (HS), which elicits anti-tumor activity by effectively modulating tumor-host interactions. We demonstrate that M402 is able to target and attenuate multiple pathways important for disease progression and that in conjunction with a cytotoxic agent is able to significantly extend the life of disease-burdened animals. Taken together, these results demonstrate that attenuating multiple extracellular signaling pathways with a heparin sulfate mimetic can have a significant effect on tumor burden and formation and growth of metastatic loci.

## Materials and Methods

### Ethics Statement

All animal experiments were conducted in compliance with the NIH Guidelines for the Care and Use of Laboratory Animals and approved by the Institutional Animal Care and Use Committee of Momenta Pharmaceuticals, Inc. Protocol # 01-2007.

### Animals, cell lines and reagents

Mice were purchased from Charles River Laboratories (Wilmington, MA).

The murine B16F10 melanoma cell line was acquired from Dr. Fidler (M.D. Anderson Cancer Center, Houston, TX). The murine 4T1 breast cancer line was purchased from ATCC (Manassas, VA). The 4T1-luc2-1A4 cells line [17] was acquired from Caliper LifeSciences (Alameda, CA) and selected for high luciferase expression and high metastatic rate by *in vivo* selection in BALB/c mice. Human T cell lymphoma Jurkat and murine leukemia WEHI-3 cells were purchased from ATCC.

Dalteparin sodium injection (10,000 IU/ml, Eisai, Inc, Ridgefield Park, NJ), Cisplatin injection solution (Bedford Laboratories, Bedford, OH) and Docetaxel injection solution (Sanofi Aventis U.S., LLC, Bridgewater, NJ) were freshly diluted before each injection.

### Preparation of M402

M402 was prepared by controlled depolymerization of unfractionated heparin in the presence of nitrous acid to yield a low molecular weight heparin having an average molecular weight of approximately 5500–6500 Daltons. This compound was further subjected to sequential periodate oxidation and borohydride reduction treatmentsand the final product was isolated and purified by salt-methanol precipitation to yield M402.

### Preparation of M-ONC 202

As described above, controlled depolymerization of unfractionated heparin was carried out in the presence of nitrous acid to yield a low molecular weight heparin with an average molecular weight of approximately 5500–6500 Daltons. The LMWH was converted to its pyridinium salt and subsequently N-desulfated following a modification of the solvolytic method as reported by Nagasawa *et. al.* [18] to yield M-ONC 202.

### Anti-coagulant activity

Plasma samples were tested for anti-Xa activity using the Chromogenix Coatest Heparin kit (Diapharma, West Chester, Ohio) on a COAG-A-MATE MTXII instrument (Organon Teknika Corporation, Durham, NC) according to manufacturers' protocols. Activated partial thromboplastin time (aPTT) was measured using the DAPTTIN TC kit (DiaPharma Group Inc, West Chester, OH).

### Surface Plasmon Resonance Assays

Surface Plasmon Resonance (SPR) assays were conducted on a Biacore T100 instrument (GE Healthcare, Piscataway, NJ). Neutravidin (Pierce Biotechnology, Rockford, IL) was immobilized on a CM4 sensor chip. Biotinylated low molecular weight heparin was captured on the sample flow cell. Growth factors including FGF2, HGF, VEGF, and SDF-1α (4, 5, 25, and 50 nM, respectively) were mixed with dilution series of each heparin sample and injected over flow cells. All data was single reference subtracted. A four parameter curve was used to fit slope versus concentration data for the standard curve. The free concentration of soluble factors in each test sample was calculated from the standard curve. The KD was calculated by the following equation:
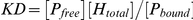
where P is protein concentration in molar units and H is the heparin concentration in mass/volume units. The reported value is the average KD calculated at three heparin concentrations.

Inhibition of the P-selectin/PSGL1 interaction by heparin-derived molecules was determined by an inhibition assay. A dilution series of each heparin-derived molecule was mixed with 25 nM P-selectin (R&D systems, Minneapolis, MN). Each mixture was flowed over the sensor surface coated with PSGL1 and the response at equilibrium was measured. The IC_50_ was calculated from equilibrium response versus concentration data by non-linear regression in GraphPad Prism. The constant of inhibition (K_i_) was calculated from the IC_50_ using the Cheng-Prushoff equation.

### Heparanase activity

Heparanase activity was measured using CisBio Bioassays technology based on time-resolved fluorescence energy transfer (TR-FRET) between europium cryptate and XL665 (allophycocyanin). The recombinant heparanase was pre-incubated with different concentrations of the heparin-derived compounds for 15 min at 37°C prior to adding to biotin-cryptate labeled heparin sulfate substrate and incubating with streptavidin-XL665 for 1 h at 37°C.

### SDF-1α/ CXCR4 chemotaxis assay

Cultured Jurkat cells (at 1×10^7^ cells/ml, 0.1 ml) were added to the upper chambers of the 3 µM chemotaxis plate (Corning Incorporated, Corning, NY). Different concentrations of LMWH were combined with 3 ng/ml SDF-1α (R&D Systems) and added to the lower chamber of a 24 well, chemotaxis plate in triplicate and incubated for 1 hr at 37°C with 5% CO_2_. Migrated cells were transferred from lower chamber of the chemotaxis plate into flow cytometry tubes and cell numbers quantified on a FACSCanto (BD BioSciences, San Jose, CA) at a fixed flow rate for 30 seconds.

### HUVEC spheroid sprouting assay

The assay was performed with a modified procedure as reported by Korff *et al.* [19]. Spheroids were prepared by pipetting HUVEC cells in a hanging drop on plastic dishes to allow overnight spheroid aggregation, before seeding in a collagen solution in the absence or presence of the test compound in a 24 well plate. Different growth factors were added on top of the polymerized gel. Plates were incubated at 37°C for 24 h and fixed by adding 4% paraformaldehyde. Images from 10 spheroids per well/data point were randomly taken using an inverted microscope. The EC sprouts with branches of each spheroid were traced manually with the digital imaging software Analysis 3.2 (Soft imaging system, Münster, Germany). All measured lengths were added to give the cumulative sprout length (CSL) of this spheroid.

### Flow cytometry analysis

Blood samples were collected by cardiac puncture. For flow cytometry analysis, 100 µl blood samples were suspended in 4 ml 1× RBC lyses buffer (eBiosciences, San Diego, CA), washed and stained with a cocktail of antibodies. All antibodies were purchased from BD Biosciences (San Diego, CA) or eBiosciences. Flow cytometry analyses were performed on a FACSCanto (BD Biosciences, San Jose, CA), and data analyzed with FlowJo (TreeStar, Palo Alto, CA).

### Murine B16F10 melanoma experimental metastasis model

Groups of female C57BL/6 mice (n = 8–13) were injected with a single dose of saline or different heparin-derived compounds at various concentrations subcutaneously, immediately followed by intravenous injection of 2×10^5^ B16F10 cells. Animals were monitored daily and body weight recorded weekly. On day 20 after tumor inoculation, animals were euthanized by CO_2_ asphyxiation and necropsy performed. Lungs were isolated weighed and then fixed in buffered formalin (VWR international, West Chester, PA). Tumor colonization to different organs was examined.

### Human C170HM2 colon carcinoma experimental metastasis model

Groups of male MF1 nude mice (n = 10) were injected intraperitoneally with 1.5×10^6^ C170HM2 human colon carcinoma cells [20]. Daily subcutaneous injection of saline or M402 at different doses was administered staring from day 1 until the experiment was terminated 35 days after tumor inoculation. At necropsy, liver tumors were excised, weighed and cross-sectional areas measured.

### Murine 4T1 mammary carcinoma orthotopic model

Female BALB/c mice were injected into the 4^th^ mammary fat pad with 4T1 or 4T1-Luc2-1A4 cells. Primary tumors were removed by surgery. Animals were monitored closely and euthanized when displaying signs of distress or until the experiment was terminated on day 32.

### Labeling of M402 with fluorescent dye

Fluorescent-labeled heparin was prepared by treating M402 with HiLyte Fluor™ 750 Hydrazide (AnaSpec, Fremont, CA) in the presence of 1-ethyl-3-(3-dimethylaminopropyl) carbodiimide (EDC, Sigma-Aldrich Chemicals, St. Louis, MO) at room temperature. The final product was isolated and purified by salt-methanol precipitation.

### Histology and immunohistology

Formalin-fixed lungs were embedded whole in paraffin and sections prepared. The slides were stained with hematoxylin and eosin (H&E). Tumor metastases were counted in one full section of the lung, tumor burden was quantified by cross-sectional tumor areas as a percentage of total lung area. Immunohistology studies were performed with formalin-fixed primary tumors and lung tissues. Standard colorimetric immunohistochemical methods were used to stain paraffin sections for CD31 using a rat monoclonal antibody (Biocare Medical, Concord, CA) and reagents required for the avidin-biotin peroxidase method for the detection of the antigen-antibody complex.

### Statistical analysis

The statistical significance of differential findings between experimental groups and controls was determined by one-way ANOVA or t-test. Log-Rank test was used for elongation of lifespan studies. Analyses were performed using GraphPad Prism software (GraphPad Software Inc., San Diego, CA). Findings were regarded as significant if P<0.05.

## Results

### Engineering of M402

**Figure 1:**
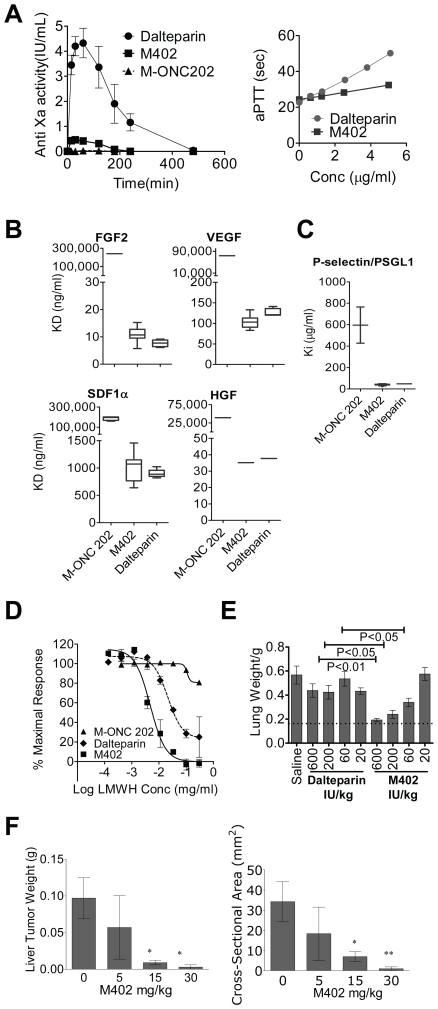
M402 displays low anticoagulation activity while retaining activity against key HSPG-binding proteins. (A) Left panel, anti-factor Xa activity: BALB/c mice were injected subcutaneously with 10 mg/kg dalteparin, M402 or M-ONC 202. Plasma was collected at different time points and analyzed for anti-Factor Xa activity (Mean±SD). Experiments were performed three times with similar results. Right panel, aPTT measured in normal human plasma. (B) The binding affinity (Mean±SD) of M402, M-ONC 202 and dalteparin to different heparin binding proteins was determined by Surface Plasmon Resonance (SPR) with a competitive inhibition assay on a Biacore T100 instrument. Different heparin-derived compounds were captured on the sensor chip with a fixed amount of growth factors including FGF2, HGF, VEGF, and SDF-1α (4, 5, 25, and 50 nM, respectively) mixed with dilution series of each heparin-derived compound flow through. The IC_50_ (µg/mL) was calculated for each interaction. (C) Inhibition of the P-selectin/PSGL1 interaction (Mean±SD) by heparin-derived molecules was determined by an inhibition assay. A dilution series of each heparin-derived compound was mixed with 25 nM P-selectin and flowed over the sensor surface coated with PSGL1 and the response at equilibrium was measured and the K_i_ was calculated. (D) Heparanase activity (Mean±SD). Heparanase activity was measured using Cisbio Bioassays technology based on TR-FRET. (E) Murine melanoma B16F10 experimental metastasis model. Groups (n = 12–13) of female mice were treated with saline or different heparin-derived compounds prior to iv inoculation of 2×10^5^ B16F10 cells. Tumor colonization to the lung was quantified by lung weight (Mean±SEM.) on day 20. Statistics were performed with One-way ANOVA using Bonferroni's multiple comparison test. (F) Human C170HM2 colon carcinoma experimental metastasis model. Groups of male MF1 nude mice (n = 10) were injected intraperitoneally with 1.5×10^6^ C170HM2 human colon carcinoma cells. Daily subcutaneous injection of saline or M402 at different doses started 1 day later until the experiment was terminated 35 days after tumor inoculation. At necropsy, liver tumors were excised, weighed (Mean±SD, left panel) and cross-sectional areas (Mean±SD, right panel) measured.

Both clinical and animal model studies indicate that heparins mediate anti-tumor activity [21], [22]. However, the ability to fully exploit the anti-tumor properties of heparin has been limited by its anticoagulant activity. Therefore, we desired to create a heparin-like compound with limited anticoagulant activity that still retained binding to key factors important in tumor growth and metastasis. Indeed, heparin-binding proteins such as VEGF, FGF2, SDF-1α, HGF and P-selectin have been shown to mediate angiogenesis, tumor and host cell trafficking, tumor cell mobility and tumor cell seeding. Based on our investigation of the structure-binding dependence of these growth factor-heparin interactions [23], we designed a lead candidate, abbreviated M402 (Table 1). M402 displayed much lower anticoagulant activity *in vivo*, as measured by plasma anti-Factor Xa activity (AUC of 56.9 min.IU/ml) compared to animals receiving an equal dose of the low molecular weight heparin (LMWH) dalteparin (AUC of 835.5 min.IU/ml) (Figure 1A, left panel). LMWH heparins, such as dalteparin, are known to have weak activity in the activated partial thromboplastin time (aPTT) assay, which is primarily a measure of anti-Factor IIa activity. As expected, M402 exhibited reduced aPTT activity compared to dalteparin (Figure 1A, right panel).

**Table 1:** Physiochemical characteristics of M402, M-ONC 202 and Dalteparin.

Characteristic	M402	M-ONC 202	Dalteparin
Chemical characteristic	Glycol split LMWH	N-desulfated LMWH	Commercial LMWH
Anti Xa (IU/mg)	<10	0	160
Anti IIa (IU/mg)	<4	0	60
Average Molecular Weight (Da)	6000	6300	6200
Polydispersity	1.5	1.4	1.3

We tested the binding affinities of M402, dalteparin and M-ONC 202 (an *N*-desulfated LMWH displaying a similar molecular weight profile and no significant anti-Xa activity, but lacking important structural features associated with growth-factor binding, Figure 1A, Table 1) to VEGF, FGF2, SDF-1α, HGF and P-selectin. Both M402 and dalteparin exhibited equivalent affinity for FGF2, VEGF, SDF-1α and HGF (Figure 1B). In contrast, M-ONC 202, consistent with its structural attributes, displayed a reduced binding to all four proteins (Figure 1B). M402 was also able to block P-selectin/PSGL interaction with a K_i_ similar to that of dalteparin, while M-ONC 202 was significantly less effective in blocking the interaction (Figure 1C). Interestingly, M402 was approximately 6-fold more potent than dalteparin in inhibiting heparanase activity, with an IC_50_ of approximately 5 µg/ml (Figure 1D). In contrast, M-ONC 202 showed poor heparanase inhibitory activity with an IC_50_ of 37 mg/ml. These results clearly indicated the successful engineering of M402 to possess substantially reduced anticoagulant activity while retaining binding activity to key heparin-binding proteins.

To determine the contribution of anticoagulation to anti-tumor activity, M402 and dalteparin were tested in the B16F10 experimental metastasis model at equivalent doses based on anti-Factor Xa activity. Lung weight at necropsy, 20 days after tumor inoculation, was used as an indication of tumor load. The lung weights of naïve animals of similar age consistently ranged within 0.17±0.02 g and the weight gain of tumor-bearing lungs correlated with both tumor nodule number and nodule size. M402 demonstrated superior anti-tumor activity to dalteparin at equivalent doses based on anti-Xa activity (Figure 1E), indicating that the antitumor activity of M402 is independent of anticoagulant activity.

Next, M402 was tested in a second experimental metastasis model involving human C170HM2 colon carcinoma cells [20] injected into the peritoneum of male MF1 nude mice. Previous studies reported that LMWHs were unable to inhibit liver colonization of i.p. inoculated colon cancer cells [24]. M402 treatment, starting 1 day after C170HM2 cell inoculation, significantly reduced the liver tumor load in a dose-dependent manner when compared to the vehicle control group (Figure 1F). Taken together, these data demonstrate the activity of M402 in experimental models of metastases.

### M402 inhibits both tumor and host cell functions *in vitro*

**Figure 2:**
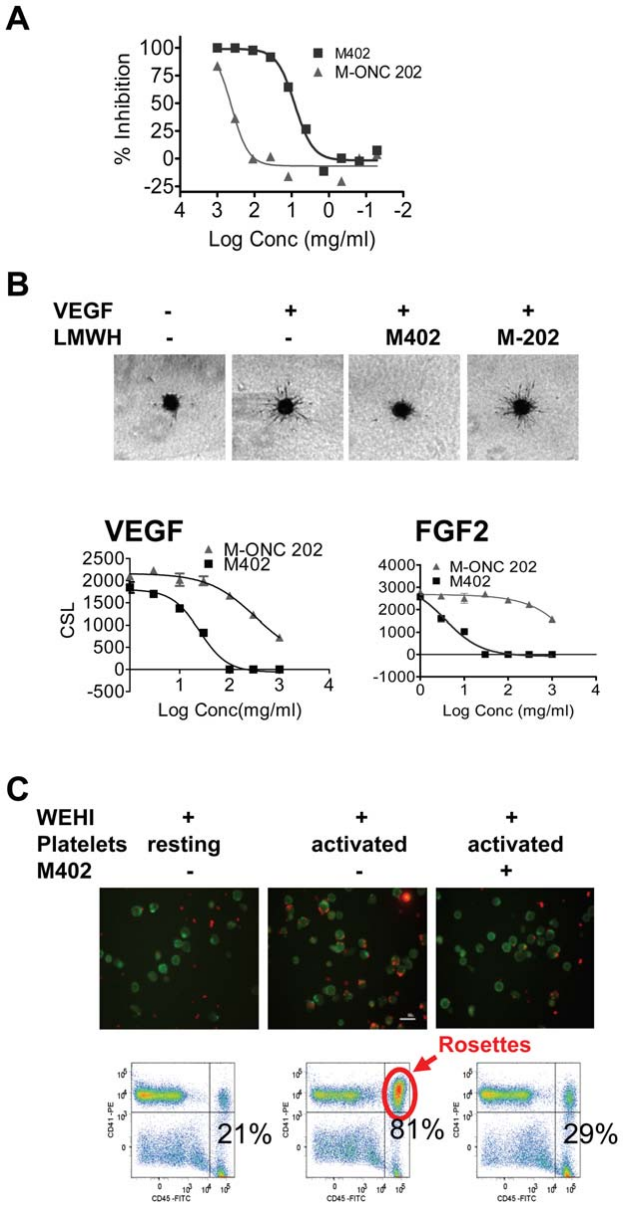
M402 inhibits tumor and host cell functions *in vitro*. (A) The SDF-1α-mediated migration assay was performed as described in Materials and Methods. Results are presented as % inhibition of migrated cell numbers. (B) M402 inhibited VEGF and FGF2 induced HUVEC sprouting. Upper panels: Representative images taken 24 hrs after incubation with VEGF-A in the presence and absence of M402 or M-ONC 202 at 30 µg/ml. Lower panels: Cumulative sprout length (CSL, Mean±SD) at different doses of M402 or M-ONC 202 in the presence of VEGF (left panel) or FGF2 (right panel). (C) M402 inhibited tumor platelet rosettes. Activated platelets were incubated for 30 min at RT with murine WEHI-3 leukemia cells (CD45^+^) pre-mixed with M402 (500 µg/ml). Platelets were stained with anti-CD41-PE and WEHI cells with anti-CD45-FITC antibodies. Platelet-WEHI-3 aggregates were imaged by immunofluorescence microscopy (upper panels) or quantitated by flow cytometry (lower panels).

Next we examined the ability of M402 to inhibit the activity of tumor or endothelial cells *in vitro*. M402 and M-ONC 202 were tested in an SDF-1α induced chemotaxis assay, using Jurkat tumor cells which were shown to express high levels of CXCR4, the cognate receptor for SDF-1α. M402 inhibited SDF-1α-induced Jurkat cell migration, with an IC_50_ of approximately 10 µg/ml, while M-ONC 202 showed greatly reduced potency in the same assay, with an IC_50_ of around 500 µg/ml (Figure 2A).

M402 was also tested in a growth factor-induced HUVEC cell spheroid sprouting assay (Figure 2B). M402 effectively inhibited sprouting of HUVEC cells in collagen gel induced by VEGF-A with an IC_50_ of 20.9 µg/ml, while the negative control M-ONC 202 had very little effect in the same assay, with an IC_50_ of around 500 µg/ml. Importantly, M402 also inhibited endothelial cell sprouting induced by FGF2 (Figure 2B), HB-EGF, and deferoxamine-induced hypoxia with IC_50_s of 5.6, 3.2, and 6.5 µg/ml, respectively.

Previous studies have shown that activation and binding of platelets to circulating tumor cells through a P-selectin-dependent mechanism facilitates seeding of tumor cells to distal tissues [15], [25]. M402 reduced the number of tumor-platelet rosettes to almost baseline levels when activated platelets were incubated *in vitro* with PSGL-expressing WEHI tumor cells (Figure 2C). This was also confirmed by *in vivo* studies in the B16F10 experimental metastasis model (see Figure 1E and results described above), a model shown to depend on P-selectin [15]. Taken together, these data indicate that M402 effectively inhibited the function of SDF-1α, VEGF, FGF2, HB-EGF, and P-selectin.

### M402 alone or in combination with chemotherapeutics demonstrates a survival benefit in the 4T1 murine mammary carcinoma orthotopic model

**Figure 3:**
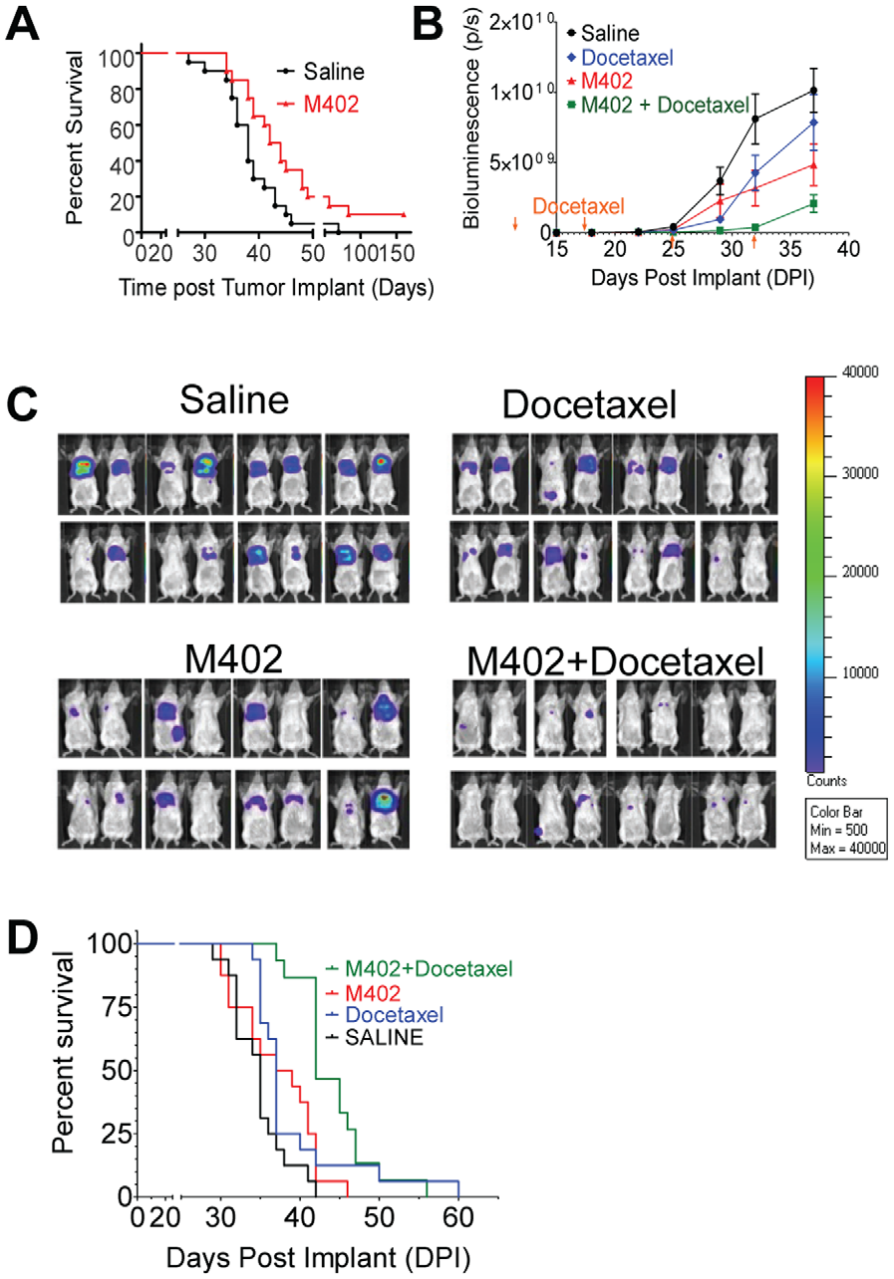
M402 monotherapy or in combination with docetaxel shows survival benefits in the orthotopic murine mammary carcinoma 4T1 model. (A) Groups of female BALB/c mice (n = 20) were inoculated orthotopically with 1×10^5^ 4T1 cells in the 4th mammary fat pad on day 0. M402 treatment delivered by sc implanted osmotic pumps at 40 mg/kg/day started on day 1. Primary tumors were removed on day 10. Survival of the M402 treated group was significantly longer than that of the saline control group (P<0.02 by Log-Rank test). (B–D) Groups of female BALB/c mice (n = 16) were inoculated orthotopically with 5×10^4^ 4T1-luc2-1A4 cells in the 4th mammary fat pad on day 0. M402 treatment delivered by sc implanted osmotic pumps at 40 mg/kg/day started on day 1. Primary tumors were removed on day 10 by surgery. Weekly ip injection of saline or docetaxel (10 mg/kg) started on day 14. After primary tumor resection, animals were monitored twice weekly with bioluminescent imaging. (B) Whole body bioluminescence (Mean±SEM) quantified as photons/second over time. (C) Bioluminescence imaging of all the experimental animals on day 29. (D) Kaplan-Meier survival curve. Survival of the M402 and docetaxel combination group was significantly longer than that of the saline control and the docetaxel monotherapy group (P<0.0001 and P<0.05, respectively, by Log-Rank test).

Given the ability to inhibit multiple pathways, we tested the efficacy of M402 in the orthotopic 4T1 murine mammary carcinoma model, an aggressive model of tumor progression and spontaneous metastasis [26]. Virtually all mice die from extensive lung metastases following resection of the primary tumor, even with treatment with conventional chemotherapeutic agents. M402 was tested first as monotherapy, and when delivered by subcutaneously-implanted osmotic pump (40 mg/kg/day), demonstrated a significant survival benefit when compared to the saline control group (P<0.02 by Log-Rank test) with 10% of the animals surviving past 160 days (Figure 3A).

We next investigated the use of M402 in combination with a standard of care cytotoxic agent. We reasoned that targeting microenvironmental factors with M402 would be complementary to targeting proliferating tumor cells with a chemotherapeutic agent. To monitor tumor progression, 4T1 cells were transfected with the luciferase gene and selected for high luciferase expression. 4T1 cells with high metastatic potential were selected by subjecting them to *in vivo* passaging [17]. Both M402 (40 mg/kg/day) and docetaxel (10 mg/kg) monotherapy moderately attenuated metastatic tumor progression, as indicated by whole body bioluminescence. M402 monotherapy showed a trend towards improved survival, but did not reach statistical significance in this study (p = 0.10 by Log-Rank test). However, the combination of M402 and docetaxel substantially inhibited metastatic tumor growth (Figure 3B and 3C) and significantly improved overall survival compared to the saline control and docetaxel monotherapy (P<0.001, and P<0.05, respectively, Log-Rank test; Figure 3D).

Notably, mice treated with M402 at 40 mg/kg/day for up to 90 days showed no significant bleeding or gross side effects such as weight loss or lethargy.

### M402 accumulates and persists in 4T1 primary tumors

M402 administered by osmotic pump at 20 mg/kg/day displayed antitumor efficacy and was shown to deliver a steady state plasma trough level of approximately 20 µg/ml. This trough level maintains M402 concentrations above the IC_50_ calculated for M402 inhibiting VEGF, FGF2, HB-EGF, heparanase and SDF-1α activity (Figure 2A and 2B). Daily subcutaneous injections of 20 mg/kg M402 showed a similar degree of efficacy in the 4T1 orthotopic model as that observed with M402 administered by osmotic pump, despite the fact that M402 has a relatively short plasma half-life of approximately 2 h (Figure 1A).

**Figure 4:**
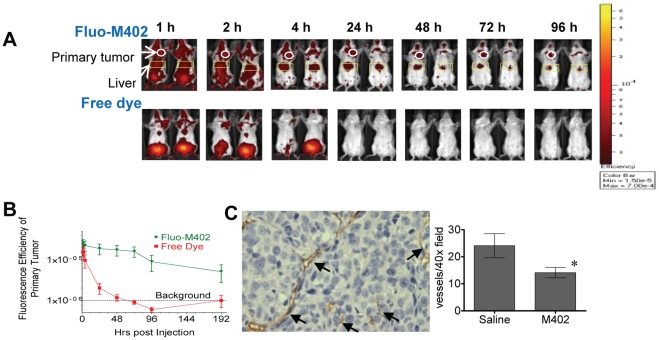
M402 targets primary tumors. Fluo-M402 biodistribution in 4T1 tumor bearing mice. Female Balb/c mice were implanted orthotopically into the first mammary fat pad at a concentration of 1×10^5^ 4T1 cells on Day 0. Mice were injected on Day 3 with a single subcutaneous dose of either 10 mg/kg fluorescently-labeled M402 or free dye of approximately the same intensity in saline. (A) Mice were imaged in the ventral view at various time points after injection with Fluo-M402 (upper panels) or free dye (lower panels). White circles indicate primary tumors, and yellow boxes highlight signals from the liver regions. (B) Quantification of fluorescent signals (Mean±SEM) at the primary tumor site at different times after Fluo-M402 or free dye injection. (C) CD31 immunohistology. Groups of female BALB/c mice (n = 16) were inoculated orthotopically with 8×10^4^ 4T1 cells in the 4th mammary fat pad on day 0. Saline or M402 (20 mg/kg/day) treatment delivered by s.c. implanted osmotic pumps started on day 5. Primary tumors were removed on day 9 by surgery and the tumor weights were recorded. There was no significant difference in primary tumor weight between the groups 4 days after the start of the treatments. Primary tumors were fixed in buffered-formalin, embedded in paraffin and stained for CD31 by immunohistochemistry. Representative CD31-staining is presented in the left panel where the brownish staining (arrows) indicate CD31^+^ vessels. Quantification of microvessel density as numbers of CD31^+^ vessels/40× field (Mean±SEM) is displayed on the right. *, P<0.05 (t-test) when compared with saline control group.

We further investigated whether M402 selectively accumulates in tumor tissue, using M402 labeled with HiLyte Fluor™ 750 dye. In normal mice, fluorescent signal was observed in the liver and bladder within 1 h after injection of labeled M402, consistent with rapid clearance of LMWH through the kidney and liver. In tumor-bearing animals, M402-associated fluorescent signals also co-localized with 4T1 cells implanted in the first mammary fat pad (Figure 4A, upper panels). Importantly, the fluorescent signal in the primary tumor area persisted up to 8 days after a single injection (Figure 4B). This observation was confirmed by *ex-vivo* imaging of isolated tumors as well as by biodistribution studies performed with ^3^H-labeled M402. In contrast, in tumor-bearing mice injected with free dye, the fluorescent signal accumulated mostly in the bladder within the first 4 h, and was not detectable 24 h after injection (Figure 4A, lower panels, Figure 4B). These results indicate that M402 accumulates and persists in tumor tissue.

### M402 reduces microvessel density in primary tumors

Based on the observed accumulation of M402 in the primary tumor, we next performed immunohistological analysis of the surgically resected primary tumors. At the time of tumor resection, which was only 4 days after the start of M402 treatment, there was no significant difference in tumor weight between the groups. However, even at this early time point, immunohistological analysis demonstrated significantly reduced CD31 staining in primary tumors from animals treated with M402 (Figure 4C), indicating reduced microvessel density in the tumor.

### M402 in combination with cisplatin inhibits tumor progression and angiogenesis at the metastatic site and normalizes circulating levels of myeloid derived suppressor cells (MDSCs)

**Figure 5:**
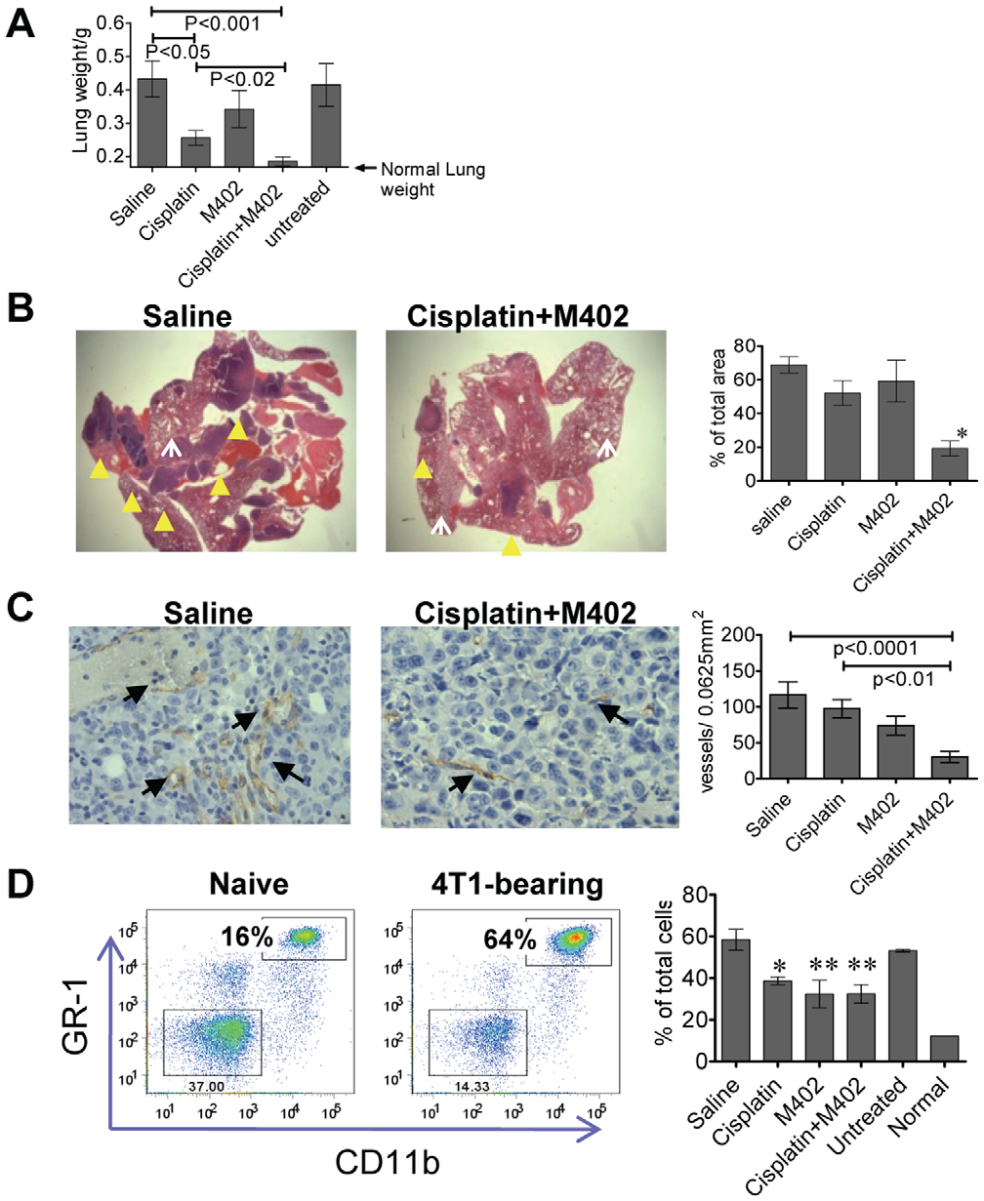
M402 inhibits tumor progression and angiogenesis at the metastastic site and normalized circulating MDSCs *in vivo*. (A) M402 combined with cisplatin inhibited lung metastasis of orthotopically inoculated 4T1 tumors. The experimental set up is described in the legand of Figure 4C. The experiment was terminated on day 32, lung tissues were isolated and lung weight (Mean±SEM) quantified (A). Fixed lungs were embedded in paraffin and % tumor (as % of total section areas, Mean±SEM) quantified under the microscope on H&E stained slides (B). Left panels show representative pictures of H&E staining where the solid darker purple stained areas (indicated by solid yellow arrow heads) represent metastatic lung tumors and the lighter-stained porous areas (indicated by white open arrows) are the normal lung tissues. Tumor areas were quantified as described in Materials and Methods, and the results are displayed in the right panel. *, P<0.05 compared to saline control, Cisplatin, and M402 monotherapy groups; one-way ANOVA. (C) Lung tissues were also stained with CD31 immunohistology. Representative images from saline and combination therapy treated lung tumors are presented in the left panel where the CD31^+^ vessels are indicated by the arrows. CD31^+^ vessels (Mean±SEM) were quantified in the tumor areas and results displayed in the right panel. Statistics were performed with One-way ANOVA using Bonferroni's multiple comparison test. (D) Blood samples obtained by cardiac puncture were analyzed by flow cytometry. Left panel: representative CD11b and GR-1 staining of blood CD45^+^ cells of naïve or 4T1-tumor bearing mice. Right panel: quantification of MDSCs as % of total cells (Mean±SEM) in different treatment groups. *, P<0.05; **, P<0.01 compared to saline control group (one-way ANOVA).

M402 was tested in combination with another standard of care chemotherapeutic, cisplatin, in the orthotopic 4T1 murine mammary carcinoma model. M402 and cisplatin therapy was initiated 5 days after tumor inoculation, around the time of onset of tumor dissemination in this model. Quantification of metastasis by lung weight (Figure 5A) or by histological evaluation (Figure 5B) demonstrated that the anti-tumor activity of M402 in combination with cisplatin was greater than that observed in the saline control, or in the cisplatin monotherapy group. CD31 immunohistology analysis was also performed on the lung tumors. Lung tumors from the cisplatin and M402 combination therapy group displayed significantly lower tumor microvessel density when compared to saline or cisplatin monotherapy groups (P<0.0001 and P<0.01, respectively; One-way ANOVA with Bonferroni's multiple comparison test; Figure 5C), suggesting the combination of cisplatin and M402 may also inhibit tumor angiogenesis at the metastatic tumor site and contribute towards reducing overall metastatic tumor load.

The expansion of CD11b^+^GR-1^+^ MDSCs has been shown to correlate with tumor progression in animal models and patients [14]. MDSCs have been implicated as angiogenic switch triggers [27] and in contributing to tumor resistance to anti-VEGF treatment [2]. M402 treatment, either as monotherapy or in combination with cisplatin, reduced metastatic 4T1 tumor-induced expansion of MDSC (Figure 5D). The reduction of circulating MDSCs in the M402 monotherapy group was similar to that observed in the combination therapy group (Figure 5D), despite the fact that the tumor load in the M402 monotherapy group was substantially greater than that of the combination therapy group (Figure 5A and B). These data suggest that M402 may directly affect MDSC expansion.

## Discussion

**Figure 6:**
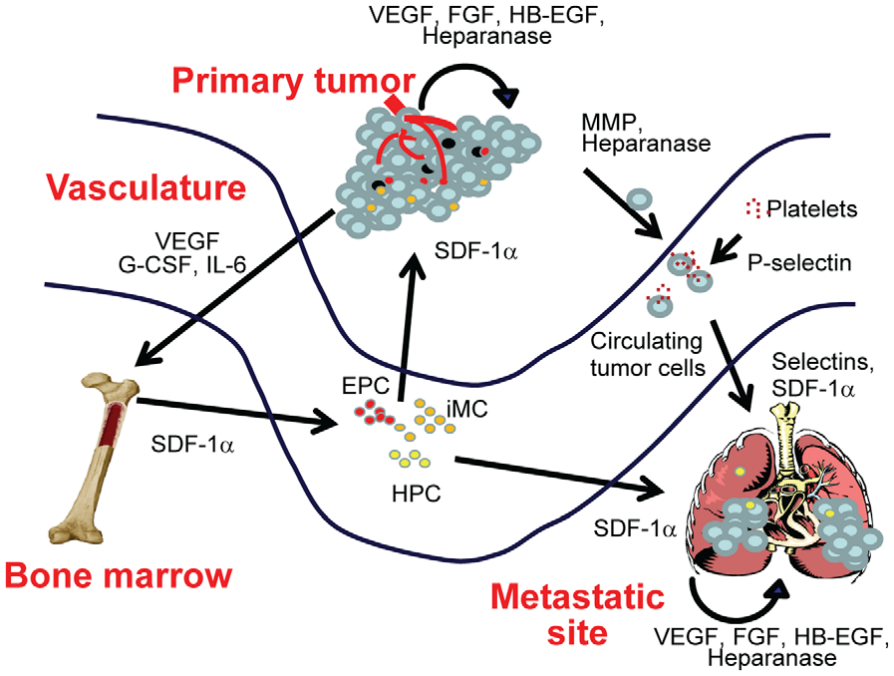
Role of HSPG-binding factors in connecting tumor niches. Abbreviations: HPC, hematopoietic progenitor cell; EPC, endothelial progenitor cell; iMC, immature myeloid cell.

Cancer is a complex disease involving changes to multiple pathways [28], [29] and microenvironments. Wels et al have proposed a complex interplay between the primary tumor, sites of metastases, and the bone marrow (illustrated in Figure 6) that is mediated by soluble factors [1]. In this model, tumor cells secrete soluble factors that can modify the microenvironment of distal tissues (e.g. sites of metastases) or induce mobilization of bone marrow-derived cells that are recruited to the primary tumor or to metastatic lesions [1]. We believe that heparan sulfate proteoglycans could play a central role in orchestrating these signals by binding and presenting growth factors and chemokines that are involved in remodeling microenvironments and recruiting distal cells. Indeed, many of the key soluble factors implicated in defining tumor-associated niches, such as VEGF, PlGF, SDF-1α, S100A8, S100A9, SAA3, TNF-α, and TGF-β [6] are HSPG-binding proteins. M402, a novel HSPG mimetic, was designed to inhibit multiple pathways involved in tumor progression and metastasis.

The anti-tumor effects of heparin or related molecules are well-documented [15], [24], [30], [31]. A number of retrospective and prospective clinical studies have indicated that heparin therapy may prolong survival of cancer patients across a variety of solid tumor types [32], [33]. Several heparin-derived molecules and synthetic carbohydrate compounds are undergoing preclinical and clinical development [34]–[36]. Despite repeated efforts, definitive data supporting anti-tumor activity of heparins in randomized clinical trials has been lacking [37] possibly because such studies have been constrained by the dose limiting anti-coagulant properties of heparin. Non-anticoagulant heparin-related molecules have been generated by various methods, such as desulfation of the 2O- and 3O-positions or *N*-desulfation followed by glycol-splitting [22], [38]. Recently, two novel heparan sulfate mimetics, PG545 and SST0001, have been described in the literature as potential anti-angiogenic/anti-metastatic agents [39], [40]. PG545 is a synthetic, fully-sulfated tetrasaccharide functionalized with a cholestanyl aglycon that shows potent inhibition of heparanase with low anticoagulant properties. PG545 exhibits a long plasma half-life and shows activity in multiple models of cancer [40]. On the other hand, SST0001 is an *N*-acetylated, glycol-split high molecular weight heparin that also exhibits low anticoagulant activity, selectively inhibits heparanase and has shown activity in an *in vivo* model of multiple myeloma [39]. However, *N*-acetylation of heparin sequences has been shown to also reduce binding affinity against many heparin-binding proteins, such as VEGF, FGF2, and SDF-1α [41]. These activities may be important for the anti-tumor activity of heparin. M402 has been specifically engineered to substantially reduce anticoagulant activity while retaining or improving its heparan sulfate-like binding properties to multiple targets involved in tumor progression and metastasis.

It is difficult to define a single ‘smoking gun’ with respect to M402's mechanism of action. This is likely due to the fact that M402 affects multiple pathways that regulate tumor cell interactions with its microenvironment. Studies investigating the mechanism of action of heparin in cancer have primarily focused on a few pathways. Folkman was the first to describe the anti-angiogenic properties of heparin, which is mediated by multiple factors including VEGF and FGF2 [9], [31], [42]. Varki and colleagues described the inhibitory effects of heparin on P-selectin-mediated cloaking of tumors cells by platelets, which promote tumor dissemination [15], [43]. Vlodavsky and colleagues have published extensively on the role of heparanase, a heparin sulfate-cleaving enzyme, in tumor progression [44]. M402 inhibits these pathways and others with similar or increased potency with respect to low molecular weight heparin, but with greatly reduced anti-Factor Xa activity.

The current armamentarium of targeted therapies aimed at specific factors, such as VEGF, have shown great promise in a variety of solid tumors. However the beneficial activity of anti-VEGF therapies, such as bevacizumab, is generally short lived. Tumors adapt by exploiting alternative angiogenic factors and recruiting EPCs and MDSCs to promote angiogenesis [2], resulting in resistance to cancer therapies and often the emergence of tumor cells with a more aggressive phenotype [3], [4], [45]. It is notable that M402 inhibits endothelial sprouting in response to FGF2 and HB-EGF as well as VEGF. In addition, M402-treated animals showed reduced numbers of circulating myeloid derived suppressor cells (MDSCs or CD11b^+^GR-1^+^ immature myeloid cells). MDSCs have been associated with tumor progression and angiogenesis [14], and may contribute to the development of tumor resistance to anti-VEGF treatment [2].The multi-targeted nature of M402 suggests the possibility that it may confer protection against the development of resistance to VEGF-specific therapies.

There has been an increased effort in identifying combinations of targeted drugs that can be used together in cancer treatments. However, combining potent single-targeted drugs is often limited by their safety profile, and can result in unexpected adverse activity [5]. Moreover the expense of combining targeted therapies has put significant pressure on the healthcare system. Targeting HSPGs is an attractive approach to targeting multiple pathways because of the versatility of HSPG functions and their importance in facilitating tumor progression [30]. Potent antibody- or small molecule-based therapies represent a digital or ‘on-off’ approach to inhibiting a specific pathway. Because of its relatively low affinity in binding to different key proteins, M402 represents an analog approach to attenuate, rather than completely shutting off, multiple pathways. This broad based attenuation of multiple pathways may confer a safety advantage over combination of current drugs that completely shut down critical pathways.

Studies interrogating heparin antitumor activity *in vivo* have been primarily restricted to experimental metastasis models wherein tumor cells are injected i.v. concurrently with a single injection of heparin. Varki and colleagues have demonstrated that heparin inhibition of P-selectin accounts for a major portion of heparin's activity in experimental models of metastasis by inhibiting platelet-tumor cell binding, which is thought to protect tumor cells in circulation and facilitate tumor cell binding to vascular endothelial cells [15], [46]. We have shown that M402 also inhibits experimental metastasis in two models, the B16F10 i.v. model and the human C170HM2 colon carcinoma model. The fact that a single dose of heparin or M402, administered concurrently with B16F10 cells, inhibits lung colonization indicates that this model primarily reflects tumor seeding. The anticoagulant properties of heparin may have constrained the ability to interrogate the effects of chronic dosing of heparin on tumor progression in spontaneous models of metastases.

To investigate the effects of M402 on metastatic processes beyond tumor dissemination, we tested M402 in a highly aggressive model of spontaneous metastasis utilizing orthotopically implanted syngeneic 4T1 mammary carcinoma cells. This model recapitulates multiple aspects of tumor dissemination, progression and clinical outcome similar to that of Stage IV human breast cancer [26]. The aggressive nature of this model is indicated by the following aspects: 1) inoculation of a single 4T1 tumor cell has the potential to develop into a tumor [17]; 2) the doubling time of the tumor cells *in vivo* is approximately 24 hours as measured by bioluminescent imaging, which means that a 75% reduction in tumor load would translate into an extension of median survival by only 2 days; and 3) standard chemotherapeutic agents show weak activity in this model. We demonstrate that M402, in combination with chemotherapeutic agents, significantly decreased metastatic tumor burden and increased survival compared to mice treated with chemotherapy alone. The effect of M402 on tumor progression beyond dissemination is reflected in reduced microvessel density, in both the primary tumor and in metastatic lesions. These results are consistent with our observation that M402 selectively accumulates in tumor tissue. Recent studies have also demonstrated the superior efficacy of M402 in combination with gemcitabine over gemcitabine alone in a genetic model (Kras^LSL-G12D/+^; p53^LSL-R172H/Flox^) of pancreatic cancer (AACR Annual Meeting 2010 Proceedings). These studies indicate that M402 affects tumor progression beyond tumor dissemination. Moreover these studies show that activity of M402, which targets multiple host microenvironmental factors, can be complementary to the direct antitumor activity of conventional chemotherapeutics agents.

In summary, our data demonstrate that targeting HSPG biology represents a potential novel strategy to treat multiple facets of cancer. Heparin, from which M402 is derived, has been used clinically for decades with an acceptable and well understood risk-benefit profile. M402 has been specifically engineered to substantially reduce anticoagulant activity while retaining or improving its other heparan sulfate-like binding properties. Here we demonstrated that M402 successfully interfered with various pathways essential for tumor progression, as well as targeted different tumor compartments. The efficacy, safety, and multimodal activity of M402 will need to be established in human clinical trials.
